# Sustained S-IgG and S-IgA antibodies to Moderna’s mRNA-1273 vaccine in a Sub-Saharan African cohort suggests need for booster timing reconsiderations

**DOI:** 10.3389/fimmu.2024.1348905

**Published:** 2024-01-31

**Authors:** Jennifer Serwanga, Violet Ankunda, Joseph Ssebwana Katende, Claire Baine, Gerald Kevin Oluka, Geoffrey Odoch, Hellen Nantambi, Susan Mugaba, Angella Namuyanja, Ivan Ssali, Peter Ejou, Laban Kato, The COVID-19 Immunoprofiling Team, Jackson Sembera, Monica Musenero, Pontiano Kaleebu

**Affiliations:** ^1^ Viral Pathogens Research Theme, Medical Research Council, Uganda Virus Research Institute and London School of Hygiene and Tropical Medicine, Uganda Research Unit, Entebbe, Uganda; ^2^ Department of Immunology, Uganda Virus Research Institute, Entebbe, Uganda; ^3^ Science, Technology, and Innovation Secretariat, Office of the President, Government of Uganda, Kampala, Uganda

**Keywords:** long-term immunogenicity, mRNA-1273 vaccine, Sub-Saharan vaccine response, S-IgG and S-IgA antibodies, vaccine-induced immunity, antibody persistence, Moderna vaccine longevity, booster dose surge

## Abstract

**Introduction:**

This study sought to elucidate the long-term antibody responses to the Moderna mRNA-1273 COVID-19 vaccine within a Ugandan cohort, aiming to contribute to the sparse data on m-RNA vaccine immunogenicity in Sub-Saharan Africa.

**Methods:**

We tracked the development and persistence of the elicited antibodies in 19 participants aged 18 to 67, who received two doses of the mRNA-1273 vaccine. A validated enzyme-linked immunosorbent assay (ELISA) was used to quantify SARS-CoV-2-specific IgG, IgM, and IgA antibodies against the spike (S) and nucleoproteins (N). The study’s temporal scope extended from the baseline to one year, capturing immediate and long-term immune responses. Statistical analyses were performed using the Wilcoxon test to evaluate changes in antibody levels across predetermined intervals with the Hochberg correction for multiple comparisons.

**Results:**

Our results showed a significant initial rise in spike-directed IgG (S-IgG) and spike-directed IgA (S-IgA) levels, which remained elevated for the duration of the study. The S-IgG concentrations peaked 14 days afterboosting, while spike-directed IgM (S-IgM) levels were transient, aligning with their early response role. Notably, post-booster antibody concentrations did not significantly change. Prior S-IgG status influenced the post-priming S-IgA dynamics, with baseline S-IgG positive individuals maintaining higher S-IgA responses, a difference that did not reach statistical difference post-boost. Three instances of breakthrough infections: two among participants who exhibited baseline seropositivity for S-IgG, and one in a participant initially seronegative for S-IgG.

**Discussion:**

In conclusion, the mRNA-1273 vaccine elicited robust and persistent S-IgG and S-IgA antibody responses, particularly after the first dose, indicating potential for long-term immunity. Prior viral exposure enhances post-vaccination S-IgA responses compared to naive individuals, which aligned with the prior-naïve, post-boost. The stable antibody levels observed post-booster dose, remaining high over an extended period, with no significant secondary rise, and no difference by baseline exposure, suggest that initial vaccination may sufficiently prime the immune system for prolonged protection in this population, allowing for potential to delay booster schedules as antibody responses remained high at the time of boosting. This finding calls for a reassessment of the booster dose scheduling in this demographic.

## Introduction

The global community experienced unparalleled disruptions due to the Coronavirus Disease 2019 (COVID-19) pandemic caused by the Severe Acute Respiratory Syndrome Coronavirus 2 (SARS-CoV-2) virus (1, 2). In response to the pandemic, joint efforts from governments, research bodies, and pharmaceutical companies rapidly advanced the development, evaluation, and distribution of vaccines as a strategic countermeasure (3). The Moderna COVID-19 vaccine (mRNA-1273) quickly emerged as a key tool in the international effort to control the pandemic (4, 5). Assessing the immune response to vaccines is vital for managing the present pandemic and anticipating future viral emergencies. Evaluating a vaccine’s immunogenicity can be screened by assessing the profiles and persistence of antibody responses, particularly the levels of IgG, IgM, and IgA (6, 7). Studies have evaluated the mRNA-1273 vaccine’s immunogenicity in different settings, using assays such as the enzyme-linked immunosorbent assay (ELISA) to detect SARS-CoV-2-specific antibodies, including IgG, IgM, and IgA (8). Despite considerable research into the effectiveness of these vaccines, key gaps in data persist, principally regarding the immune response to the mRNA-1273 vaccine in Sub-Saharan Africa (SSA). Understanding the global immunological landscape is imperative, especially since Sub-Saharan African (SSA) populations often exhibit distinct vaccine response profiles (9–11). Additionally, interpretation of immunological responses must consider an individual’s prior virus exposures, which can markedly affect antibody dynamics (12). Genetic differences, health conditions, other present infections, and nutritional and socio-economic disparities impact individual vaccine responses (13). The importance of discerning this distinct antibody response profile is paramount, focusing on temporal dynamics of vaccine-induced IgG, IgM, and IgA antibodies, post-vaccination.

This research delineated the immunological response to the mRNA-1273 COVID-19 vaccine within a Sub-Saharan African cohort, tracking and quantifying SARS-CoV-2-specific antibodies (IgG, IgM, IgA) for 12 months post-vaccination. The aim was to elucidate the vaccine-induced antibody profiles crucial for understanding immunity in this demographic. Prior exposure to the virus can influence the immune response to vaccination, sometimes resulting in detectable antibody levels that may modify subsequent immunogenicity. However, evidence also points to reduced vaccine responses in some instances (14), making investigating pre-vaccination immune status critical. The persistence of m-RNA-elicited antibody responses within the African demographic over extended periods remains a critical yet uncharted dimension of immunological research.

## Materials and methods

### Study population and design

**Figure 1:**
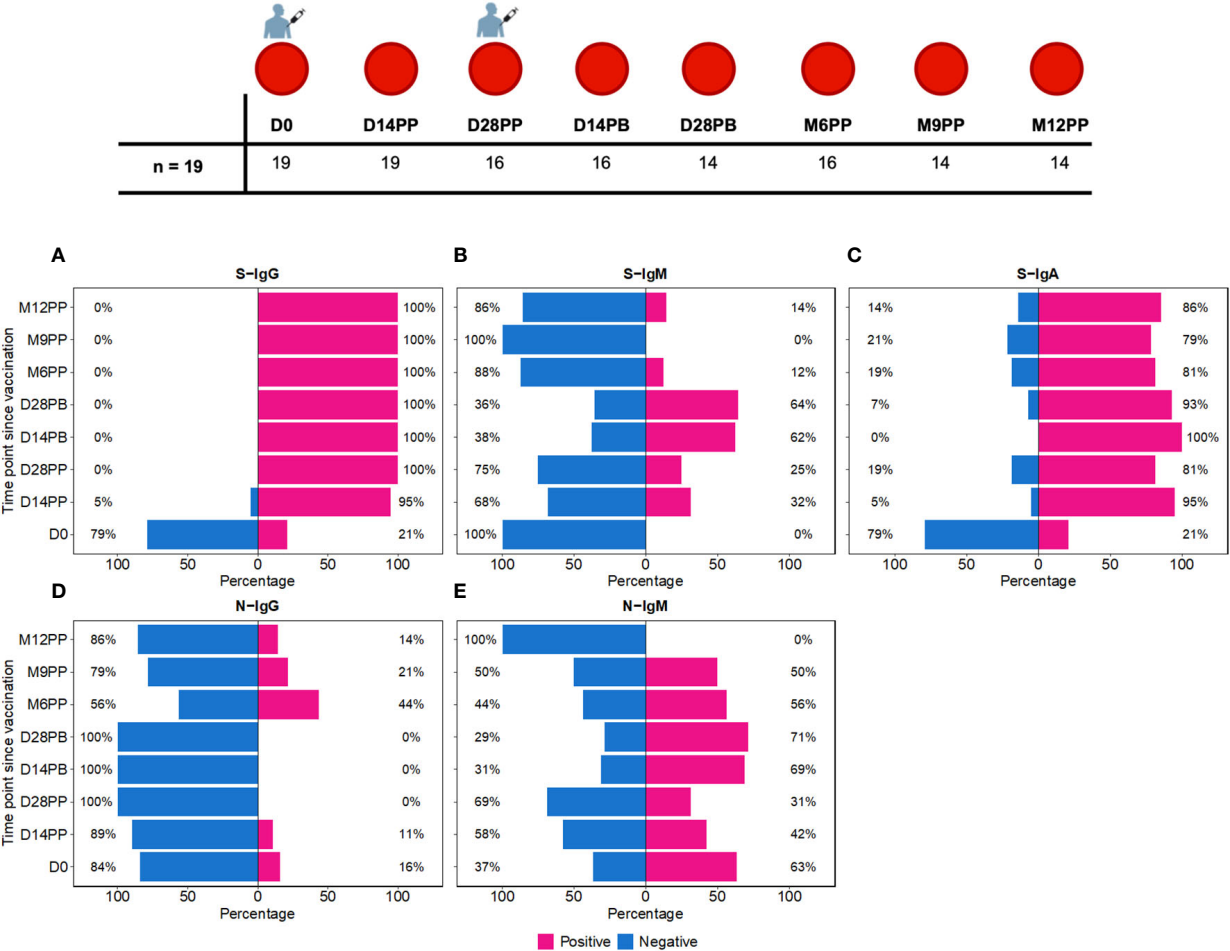
Time-Dependent Dynamics of Seroconversion Targeting Spike (S) and Nucleoprotein (N) Antigens. **Figure 1** shows the temporal progression of seroconversion post-Moderna vaccination. Baseline samples were taken right before the initial vaccination (Day 0, D0). We then collected samples at 14 (D14PP) and 28 days (D28PP) post-primary dose, and again at similar intervals after the booster dose (14 days, D14PB; 28 days, D28PB). To track long-term immunity, further samples were gathered at 6 (M6PP), 9 (M9PP), and 12 months (M12PP) following the primary dose. This figure illustrates the percentage of subjects who seroconverted over 12 months following the first dose of the Moderna vaccine, spanning eight follow-up time points. **(A–C)** depict seroconversion for Spike-directed IgG, IgM, and IgA antibodies, respectively, while **(D, E)** illustrate seroconversion for nucleoprotein-directed IgG and IgM. At each time point, subjects are categorised as either seropositive (represented in pink) or seronegative (represented in blue). The x-axis enumerates the proportions of subjects within each serostatus category, while the y-axis represents the chronological time points post-vaccination.

The study cohort comprised 19 individuals, ranging from 18.0 to 67.0 years of age, with a median age of 26.0, clustered within an interquartile range (IQR) of 23.0 to 32.5 years. Six of these participants were female, comprising 31.6% of the sampled population, while the remaining 13 were male, representing 68.4%. Participants in the study were administered two doses of the Moderna mRNA-1273 COVID-19 vaccine, receiving the first dose on day 0 and the booster between 28 to 30 days. Over 12 months, between 08-09-2021 and 22-09-2022, we collected 128 blood samples at predetermined intervals to evaluate the immunogenicity elicited by the vaccine. Baseline samples were collected immediately prior to administration of the primary vaccine dose, a time point designated as Day 0 (D0). Subsequent follow-up specimens were obtained on Days 14 (D14PP) and 28 (D28PP) after the primary dose, to assess the immediate immune responses, as depicted in **Figure 1**. After the booster dose, additional samples were collected on days 14 (D14PB) and 28 (D28PB) to evaluate the immediate immunological response post-boost. Further assessments were conducted at six (M6PP), nine (M9PP), and twelve months post-prime (M12PP) to monitor the durability of the vaccine-induced immunity.

### Binding antibody ELISA to detect SARS-CoV-2-specific IgG, IgM, and IgA levels

A validated ELISA (15, 16) was used to accurately detect and quantify SARS-CoV-2-specific IgG, IgM, and IgA antibodies against the spike and nucleocapsid proteins within our specimens, characterizing the immune response to the virus in the study population. ELISA plates were optimally coated with 3 μg/ml of antigen, a concentration validated for maximal specificity and sensitivity. Optical densities (OD) were measured at 450 nm to quantify antibody concentrations, expressed in nanograms per milliliter (ng/ml). In a prior study, we determined the optimal optical density (OD) cutoff values for seropositivity using receiver operating characteristics (ROC). This method was deemed to be the most effective of four methods tested for setting these thresholds in our population. Briefly, we used PCR-positive longitudinal samples at peak antibody levels as positive controls and pre-pandemic samples as negatives. The cutoffs were set to maximize sensitivity and give priority to specificity, adhering to the principle of minimizing the Error Rate (ER) function (*c*). The area under the curve (AUC) was also evaluated to gauge overall effectiveness of these cut-offs. To validate our established cutoff values, we applied them to distinguish between negative and positive samples in the WHO anti-SARS-CoV-2 verification standards panel WHO 20/B770-02 S-IgG. This validation yielded 100% specificity and 100% sensitivity, confirming the reliability of our cutoff values. These procedures have been detailed in our assay optimization publication (16). In this study, seropositivity was determined using previously established cut-off OD values for this population, which are 0.432 for IgG, 0.459 for IgM, and 0.226 for IgA against spike-specific antibodies, and 0.454 for IgG, 0.229 for IgM, and 0.225 for IgA for nucleoprotein-specific antibodies. In our previous research (16), we established that an anti-spike IgG antibody threshold of 0.432 corresponds precisely to 18.94 binding antibody units (BAU)/ml.

Our ELISA assay directly measured OD at 450nm and antigen concentrations in ng/ml, using commercial IgG, IgM, and IgA standards that we calibrated against WHO standards to generate BAU/ml units. Due to the assay’s intrinsic design to measure ng/ml and the limited availability of WHO standards, it was not feasible to generate direct BAU/ml measurements. The BAU/ml values in **Table 1** stem from a robust conversion model analysis detailed before (16). For consistency, all primary concentration data here will be precisely reported in ng/ml, which were directly determined by the assay. Corresponding binding BAU/ml computed values are detailed in **Table 1** for reference.

**Table 1:** Longitudinal Analysis of S-Antibody Optical Densities and Concentrations across Predefined Time Points in the Study Cohort.

Time Point	Antibody	Median OD (IQR)	Median Conc (ng/ml)	Median Conc (BAU/ml)
D0	S-IgG	0.125 (0.047, 0.382)	540.5 (138.9, 2170.3)	7.686 (2.203, 33.530)
	S-IgM	0.098 (0.061, 0.125)	300.2 (215.9, 347.3)	11.298 (7.750, 13.259)
	S-IgA	0.061 (0.011, 0.163)	482.2 (151.95, 1524.85)	91.992 (28.964, 290.983)
D14PP	S-IgG	0.953 (0.755, 1.300)	36590.15 (5729.05, 120749.20)	685.377 (107.380, 2261.592)
	S-IgM	0.295 (0.230, 0.516)	875.5 (552.95, 1620.15)	32.76844 (20.867, 60.244)
	S-IgA	1.083 (0.677, 1.530)	6537.5 (3272.90, 13389.15)	1247.652 (624.600, 2555.297)
D28PP	S-IgG	0.820 (0.729, 0.933)	54241.6 (19522.7, 137972.0)	1015.971 (365.721, 2584.158)
	S-IgM	0.239 (0.137, 0.417)	729.95 (316.475, 1141.025)	27.39808 (12.142, 42.566)
	S-IgA	0.809 (0.442, 1.149)	4243.6 (1894.275, 8015.300)	809.8593 (361.488, 1529.692)
D14PB	S-IgG	0.839 (0.721, 1.179)	157407.2 (92881.57, 252734.80)	2948.16 (1739.659, 4733.550)
	S-IgM	0.539 (0.275, 0.729)	1800.85 (655.950, 2462.775)	66.911 (24.668, 91.334)
	S-IgA	1.188 (0.905, 1.515)	8139.7 (5086.00, 13836.85)	1553.434 (970.632, 2640.741)
D28PB	S-IgG	0.801 (0.751, 1.089)	88367.6 (64690.12, 202010.40)	1655.117 (1211.662, 3783.533)
	S-IgM	0.504 (0.425, 0.580)	1802.8 (900.400, 2294.875)	66.98303 (33.687, 85.139)
	S-IgA	1.001 (0.554, 1.249)	6086 (2653.1, 10750.9)	1161.483 (506.311, 2051.784)
M6PP	S-IgG	0.978 (0.710, 1.161)	53240.0 (25211.70, 69643.85)	997.212 (472.270, 1304.440)
	S-IgM	0.258 (0.190, 0.383)	732.6 (507.750, 1301.925)	27.496 (19.200, 48.502)
	S-IgA	0.900 (0.358, 1.054)	4859.9 (1430.10, 6364.15)	927.481 (272.900, 1214.568)
M9PP	S-IgG	1.129 (0.818, 1.200)	38431.2 (20799.6, 52933.8)	719.858 (389.636, 991.478)
	S-IgM	0.215 (0.167, 0.347)	660.95 (500.325, 1149.100)	24.8521 (18.9256, 42.8635)
	S-IgA	0.796 (0.324, 0.957)	4356.3 (1338.575, 5621.400)	831.368 (255.432, 1072.814)
M12PP	S-IgG	1.215 (1.060, 1.238)	48217.35 (25417.5, 58195.8)	903.143 (476.125, 1090.030)
	S-IgM	0.236 (0.142, 0.327)	868.0 (512.025, 1228.950)	32.492 (19.357, 45.810)
	S-IgA	0.660 (0.503, 0.763)	2857.7 (1976.750, 3541.225)	545.3589 (377.229, 675.810)

**Table 1** presents optical densities (OD) at 450 nm and the corresponding concentrations in nanograms per millilitre (ng/ml) for IgG, IgM, and IgA antibodies at each specified time point. The ELISA directly quantified these parameters, using a commercial standard, which was calibrated against WHO standards to derive Binding Antibody Units (BAU) per ml (BAU/ml), using a conversion model analysis, as described before (16).

### Statistical methods

In our cohort, antibody responses were quantitatively compared at multiple time points using box plots to depict the medians (horizontal lines), means (dots), and interquartile ranges (top and bottom edges of the box), while diverging bar graphs were used to show the percentage of participants undergoing seroconversion. To identify significant temporal changes in antibody levels, we employed the Wilcoxon test for pairwise comparisons, with the Hochberg correction applied to address the potential for type I errors due to multiple testing. We utilized unpaired statistical tests to accommodate the intermittent absence of samples/data at various time points. P-values above 0.05 were considered non-significant, denoted as “ns”, while levels of significance were marked as follows: * (p ≤ 0.05), ** (p < 0.01), *** (p < 0.001), and **** (p < 0.0001).

## Results

### Dynamic patterns of seroconversion and antibody dynamics post-vaccination

We observed distinct seroconversion patterns over time following vaccination. Initially, only 21% of 19 subjects exhibited S-IgG seropositivity, but this dramatically increased to 95% by 14 days post-priming (D14PP) and reached 100% by day 28 post-priming (D28PP), with sustained high S-IgG seropositivity thereafter [**Figure 1A**]. In contrast, all subjects were S-IgM negative at baseline (D0). However, 32% developed S-IgM responses by D14PP, peaking at 64% by day 28 post-boost (D28PB) before declining to zero by month nine post-priming (M9PP) [**Figure 1B**]. Regarding S-IgA, we noted a rise from 21% pre-vaccination to 95% by D14PP, with approximately 80% maintaining elevated levels throughout the study [**Figure 1C**]. In contrast, N-IgG responses were initially low at 16%, diminishing to zero by D28PB. However, a resurgence was observed post-D28PB, with 44% of subjects developing N-IgG responses. N-IgM responses were observed in approximately half of the subjects until month 12 post-priming (M12PP) when all subjects became N-IgM seronegative [**Figure 1D**]. These findings underscore the dynamic and robust immunological responses elicited by the vaccine over the study period, characterized by a rapid and sustained increase in S-IgG and S-IgA seropositivity, a transient rise in S-IgM, and a delayed but significant emergence of N-IgG antibodies.

### S-IgG and S-IgA antibody levels showed a sustained presence over 12 months, while S-IgM levels were transient

Following the initial mRNA-1273 vaccination, S-IgG antibody levels exhibited a pronounced increase, with OD values at 450 nm soaring from a baseline of 0.125 (IQR 0.047–0.382) to 0.953 (IQR 0.755–1.303) by day 14, with corresponding concentrations in ng/ml rising from 540.5 (IQR: 138.9, 2170.3) to 36590.2 (IQR: 5729.1, 120749.2). This heightened response was maintained at day 28 post-priming, with median S-IgG OD values at 0.820 (IQR 0.729–0.933) and concentrations reaching 54241.6 ng/ml (IQR 19522.7, 137972.0). After the booster dose, a nearly three-fold rise in S-IgG antibody concentrations was observed by the 14th day, peaking at 157407.2 ng/ml (IQR 92881.6, 252734.8) by day 14; however, this initial increase did not achieve statistical significance when adjusted for multiple comparisons. Sustained S-IgG elevated levels were noted after that, with concentrations of 53240.0 ng/ml (IQR 25211.70, 69643.85) at six months and 38431.2 ng/ml (IQR 20799.6, 52933.8) at nine months post-primary dose, as seen in **Table 1**.

**Figure 2:**
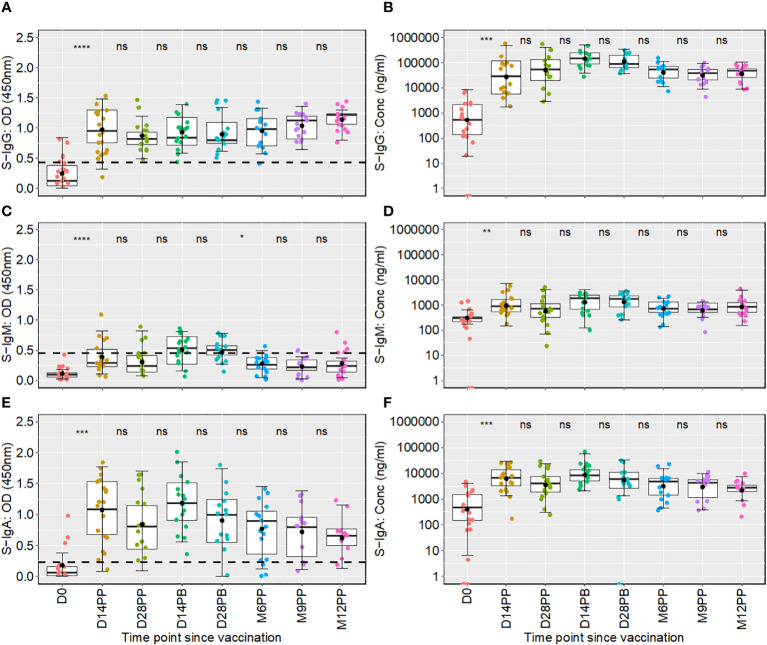
Boxplots showing longitudinal distributions of spike-directed antibody optical densities (OD) and concentrations across a 12-month follow-up period. Figure 2 shows the temporal distribution of Spike-IgG **(A, B)**, Spike-IgM **(C, D)** and Spike IgA **(E, F)** antibody responses over 12 months following the initial Moderna vaccine dose. Each box plot displays the interquartile range, with the mean represented by a solid black circle and the median by a horizontal line within the box. Differences in antibody responses between time points were evaluated using the unpaired Wilcoxon test, with the Hochberg test applied for multiple testing correct. The x-axis on the graph denotes the time elapsed since vaccination, with “PP” indicating the time points after the initial vaccine dose (post-prime) and “PB” representing the intervals following the booster dose (post-boost). Meanwhile, the y-axis measures the optical densities and concentrations detected. The comparative analysis of significance was conducted between successive time points to evaluate the temporal evolution of antibody responses within the study. This methodological choice ensured that the significance is attributed to changes observed between consecutive time points. Consequently, significance values are presented between these time points, rather than above a box plot, to reflect their temporal association. Significance levels are indicated as not significant ns, p > 0.05; *, p ≤ 0.05; **, p < 0.01; ***, p < 0.001 and ****, p < 0.0001.

One year after the initial vaccination, median S-IgG levels rose to high levels of 48217.35 ng/ml (IQR 25417.5, 58195.8), suggesting potential reinfections. However, after adjustments for multiple comparisons, statistical analyses at the various timepoints did not reveal significant differences in S-IgG concentrations at any time point beyond the initial significant surge two -weeks post-vaccination, as depicted in **Figures 2A, B**. While S-IgM levels marginally surpassed the cut-off briefly after the booster they subsequently waned, contrasting with S-IgA, which significantly increased from 482.2 to 6537.5 ng/ml within two weeks of the first dose (p<0.001, Wilcoxon unpaired test with Hochberg correction), and maintained above-threshold levels thereafter, as seen in **Figures 2E, F**. Meanwhile, nucleoprotein-directed IgG (N-IgG) and nucleoprotein-directed IgM (N-IgM) showed negligible variability, as shown in **Supplementary Figure 1**.

Taken together, the mRNA-1273 vaccine induced a robust S-IgG antibody response, which persisted for at least one year, suggesting the potential for long-term immunity. In contrast, S-IgM responses appeared transient and waned over time. A notable and persistent increase in S-IgA antibodies postulates the latter’s likely role in ongoing immune protection against the virus.

### Fold changes in antibody responses showed substantial elevations in antibody concentrations, especially 14- and 28-days post-boost

**Figure 3:**
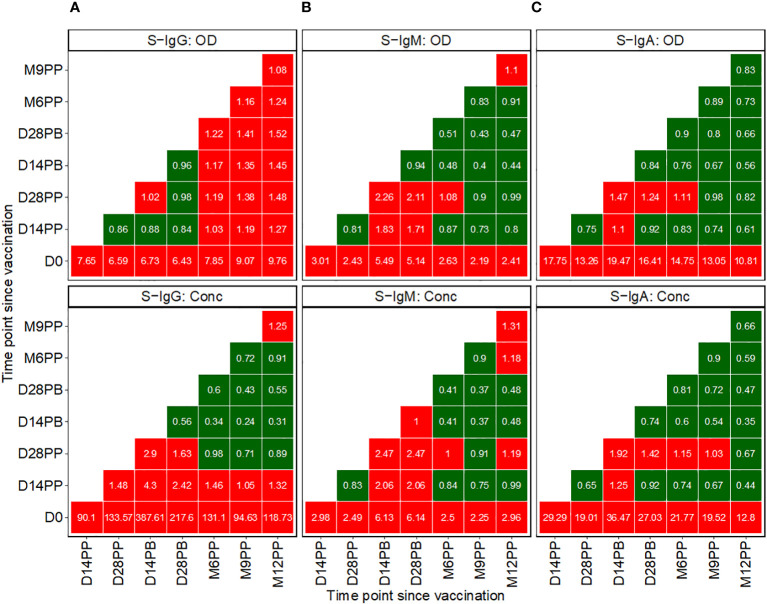
Pairwise Temporal Dynamics of Median Fold-Changes in Spike-Directed Antibody Responses Across Sequential Time Points. **Figure 3** illustrates the median changes in Spike-directed IgG **(A)**, IgM **(B)**, and IgA **(C)** antibodies over time. The fold change quantifies the ratio of median antibody levels between a reference timepoint (on the y-axis) and a subsequent timepoint (on the x-axis). Red boxes indicate increases, while green boxes indicate decreases. A value of one in a box signifies no change from the baseline reference, values below one indicates decreases, and values above one represents increases.

Subsequent analysis examined the median changes in antibody OD levels and concentrations across successive time points, as presented in **Figure 3**, with increments marked in red and decrements in green. Compared to pre-vaccination levels, we noted a consistent and significant fold-rise in spike-directed IgG antibody OD, to at least six-fold across all time points. The most substantial fold-rise occurred at 12 months, registering a 9.76-fold increase in OD levels compared to baseline. Correspondingly, S-IgG antibody concentrations also surged significantly, peaking to 387.6 times above the baseline at day 14 and maintaining at 217.7 fold at day 28 post-boost. After boosting, a modest rise in antibody concentrations occurred, registering 2.9 times the baseline at day 14 and 1.6 times at day 28, before eventually diminishing, as detailed in **Figure 3A**.

We observed a modest rise in IgM levels, achieving at least a two-fold increase from baseline following vaccination. The most pronounced IgM elevations were detected at two- and four-weeks post-boost relative to baseline, with OD levels rising by 5.49- and 5.14-fold and concentrations by 6.13- and 6.14-fold, respectively. In contrast, at 14 and 28 days after the booster, IgM OD levels showed increases of 2.26 and 2.11 times, respectively, and concentrations increased by factors of 2.47 at both time points, as detailed in **Figure 3B**.

Following initial vaccination, IgA levels exhibited the most significant fold change among all isotypes, with OD fold changes ranging between 10.81 and 19.47. Specifically, we observed a 17.75-fold increase in IgA OD levels two weeks post-prime and a 13.26-fold increase four weeks post-prime compared to baseline. Post-booster rises were comparatively moderate, with a 1.47-fold increase at 14 days and a 1.24-fold increase at 28 days. In terms of antibody concentrations, IgA levels rose substantially, up to 29.29-fold at 14 days and 19.01-fold at 28 days post-prime. Following the booster, the IgA concentration elevations were more subtle, with a 1.92-fold increase at the two-week mark and a 1.42-fold increase at four weeks, before reverting to pre-boost levels, as depicted in **Figure 3C**. Changes in S-IgM concentrations were modest across the study’s timeline. The study recorded only minor variations in N-IgG and N-IgM levels, as shown in **Supplementary Figure 2A**. In this population, an 11-fold increase in N-IgG concentration relative to the prior timepoint is indicative of an infection (6). This criterion was used to identify breakthrough cases. An 11-fold elevation in N-IgG levels 14 days post-complete vaccination was indicative of infection, qualifying subjects as breakthrough cases. Analysis of the data six months following complete vaccination revealed three such breakthrough infections. Of these, two subjects were seronegative for S-IgG at baseline, while one was seropositive, as summarized in **Supplementary Figure 2B**.

### Baseline S-IgG serostatus impact on post-vaccination S-IgG and S-IgA dynamics

**Figure 4:**
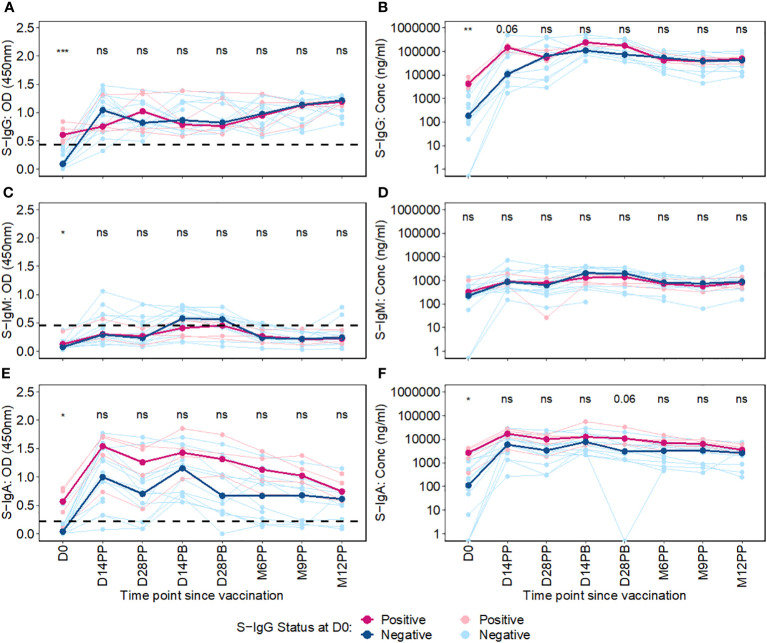
Longitudinal Trajectories of Median S-Protein Antibody Responses Stratified by Baseline S-IgG Status. Figure 4 shows the longitudinal trends of SARS-CoV-2-specific Spike-IgG **(A, B)**, Spike-IgM **(C, D)** and Spike IgA **(E, F)** antibody responses stratified by baseline S-IgG seropositivity. Participants were grouped based on their S-IgG OD levels at D0. Those with S-IgG levels greater than or equal to the cut-off (0.432 OD) were designated as baseline S-IgG+ (illustrated by red lines), while those with levels below this threshold were termed baseline S-IgG- (represented by blue lines). The x-axis indicates the follow-up duration postseroconversion, while the y axis displays the antibody optical densities at 450 nm alongside the corresponding concentration in ng/ml. In this figure, individual data points are represented: S-IgG positive cases are marked in light red dots, while S IgG negative cases are shown in light blue dots. Median antibody levels for each category are shown with darker tones of red and blue. In the figure, median antibody responses between baseline S-IgG+ and S-IgG- subjects at each time point are compared using an unpaired Wilcoxon test. Significance levels are indicated as not significant ns, p > 0.05; *, p ≤ 0.05; **, p < 0.01, and ***, p < 0.001.

We then used an unpaired Wilcoxon test to examine the differential antibody responses between baseline S-IgG+ and S-IgG- subjects at various time points, as depicted in **Figure 4**. Subjects were categorized based on baseline S-IgG levels, with those equal to or above the cutoff 0.432, equivalent to 18.94 BAU/ml were deemed S-IgG+ and the rest as S-IgG-. Initially, at Day 0, S-IgG+ subjects exhibited significantly elevated S-IgG antibody levels compared to their S-IgG- counterparts. Following the priming dose, both groups experienced a marked increase in S-IgG antibody responses within the first 14 days. Notably, this rise was more pronounced in the S-IgG- group. However, by Day 14 post-prime, the differences in antibody levels between the two groups were not statistically significant, although a trend toward higher levels in the S-IgG+ group was observed. Beyond Day 14 post-prime, S-IgG responses did not significantly differ between the groups, as shown in **Figures 4A, B**. In contrast, S-IgM antibody responses remained relatively low throughout the study period, with optical density (OD) levels mostly below the cutoff for both groups, indicating no significant differences between S-IgG+ and S-IgG- subjects (**Figures 4C, D**). Regarding S-IgA antibodies, S-IgG+ subjects started with significantly higher OD and concentration levels above the cutoff compared to S-IgG- subjects who began below the cutoff. Both groups showed a substantial increase from Day 0 to Day 14 post-prime with no significant difference across all follow-up time points between the groups, except for a tendency towards higher S-IgA levels in the S-IgG+ group 28 days following priming, as illustrated in **Figures 4E, F**.”

Overall, this study showed initial elevation of S-IgG antibodies post-vaccination across all participants, within the first 14 days regardless of baseline anti-spike serostatus, particularly evident within the S-IgG negative group. While the antibody-naive S-IgG- group experienced a more pronounced S-IgG response, levels eventually converged with the pre-exposed S-IgG+ group, and stayed similarly elevated throughout follow-up, with no significant difference across groups.

### Comparative analysis of antibody responses post-primary and booster vaccination stratified by baseline spike-IgG status

**Figure 5:**
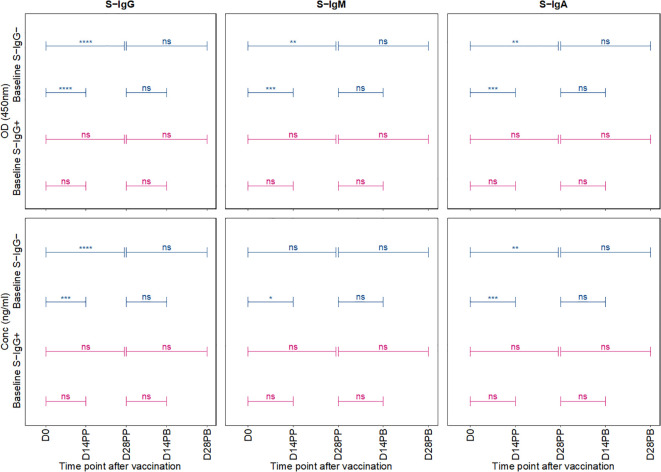
Comparative Analysis of Antibody Responses Post-Primary and Booster Vaccination Stratified by Baseline Spike-IgG Status. **Figure 5** presents a comparative analysis of antibody optical densities (OD) and concentrations (ng/ml) following primary and booster immunizations, segregated by initial Spike-IgG levels, key data for these comparisons is shown in earlier plots. Participants are categorised into Spike-IgG positive (red) and negative (blue) groups. The figure illustrates changes in Spike-specific IgG, IgM, and IgA antibody levels at two and four weeks after each vaccine dose. The significance of the difference in antibody responses after each dose is evaluated using an unpaired Wilcoxon test, with multiple testing adjustments made using the Hochberg method. Significance levels are denoted as not significant ns, p > 0.05; *, p ≤ 0.05; **, p < 0.01; ***, p < 0.001, and ****, p < 0.0001.

In a comparative analysis of antibody responses following the primary and booster doses, stratified by baseline spike protein-specific immunoglobulin G (S-IgG) status, distinct patterns emerged. Among the initially S-IgG positive subjects (n = 4 with 31 samples), no significant difference occurred in spike-directed antibody OD levels and concentration of across the pairwise time points. However, this finding must be interpreted with caution due to the limited sample size of only four subjects in the S-IgG+ group. In contrast, subjects without baseline S-IgG demonstrated a notable increase in spike-directed antibody responses between day 0 (D0) and day 14 post-primary dose (D14PP), and between D0 and day 28 post-primary dose (D28PP). Interestingly, after boosting, these significant differences did not hold (**Figure 5**). Overall, these results show some differences in antibody responses based on baseline S-IgG status and highlight the influence of prior immunity on subsequent vaccine-induced immune responses.

## Discussion

The novel SARS-CoV-2 virus presented significant global challenges to health infrastructures and economies. This catalyzed extensive research into potential vaccines. The swift development, deployment, and administration of COVID-19 vaccines highlighted the global response to the pandemic. Among these, Moderna’s mRNA-1273 vaccine demonstrated substantial efficacy in diverse global settings (17, 18), but little is known about its performance in the SSA setting. While performance of the mRNA-1273 vaccine has been extensively studied in various global settings, its performance within the SSA context remains scant. The genetic, microenvironments, and antigenic exposures distinctions of the SSA population, known to affect vaccine responses underscore the importance of evaluating the vaccine in this unique milieu. The distinct demographic landscape, influenced by a confluence of factors known to affect vaccine responses, including genetic (19–21), racial (22) environmental, and socio-economic determinants (23, 24) highlight the imperative to evaluate the efficacy of the mRNA-1273 vaccine in the context of the SSA milieu, especially given that varied ethnic groups within the same region have demonstrated differential vaccine responses and antibody decay rates, pointing towards a genetic influence on vaccine immunogenicity (25, 26). Here, we sought to comprehensively evaluate the dynamics and long-term immunogenicity of the mRNA-1273 vaccine and its capacity to maintain persistent immune responses within this demographic.

Our study revealed notable dynamics in antibody responses following both doses of the Moderna mRNA-1273 vaccine. A significant and sustained presence of S-IgG and S-IgA antibodies was noted over 12 months, while S-IgM antibodies were more transient. After the primary dose, a significant elevation in S-IgG antibody levels was observed, with the concentration peaking 28 days post-boost. While there was a marked rise in S-IgA levels post-priming, S-IgM levels remained suboptimal and fell below the designated threshold over time. Participants categorized based on their S-IgG levels at baseline demonstrated variations in S-IgA antibody dynamics throughout the study, with those previously exposed to the virus (S-IgG+ group) consistently showcasing greater S-IgA antibody responses. Our findings concur with previous studies indicating that prior viral exposure enhances the potency of the subsequent IgA response following vaccination compared to infection naïve individuals (27, 28). The substantial increase in S-IgG and S-IgA antibodies following the first vaccine dose is a positive outcome, as high levels have been shown to correlate with both protection and long-term persistence. Elevated serum monomeric S-IgA levels may indicate the shedding of dimeric IgA into mucosal areas. Since both IgA forms possess neutralizing capabilities, their presence is indicative of potential protective immunity (29). Global studies have demonstrated a strong inverse correlation between binding and neutralizing antibody markers and the risk of COVID-19 infection while simultaneously establishing a direct correlation with the efficacy of mRNA COVID-19 vaccines (30). This evidence contributes significantly to establishing immune markers as surrogate endpoints for assessing the effectiveness of these vaccines (31, 32). These sustained antibody levels, particularly for S-IgG, suggest prolonged immunity, a crucial characteristic for any successful vaccination program (33).

The transient nature of S-IgM aligns with its known biological role as an early responder, which wanes as the immune system transitions to produce more lasting and specific antibodies like IgG (34–36). The elevated S-IgA responses, especially in the S-IgG+ group, highlight the mucosal immunity’s potential role. Since S-IgA is a principal player in mucosal immunity, its sustained presence in the prior exposed aligns with other cohorts (37, 38), and the lower responses in prior naïve agrees with prior data (28), suggesting the vaccine’s potential in mitigating respiratory tract infections (38), a predominant site for SARS-CoV-2 entry.

The absence of a significant response post-booster raises questions. It could be inferred that a single dose might provide substantial protection in this demographic for a substantial duration, reducing the necessity of a booster one month after the first dose. Alternatively, it might suggest a ceiling effect, where maximal immune stimulation is already achieved post-priming, rendering the booster less effective in further enhancing immunity at that timing. Studies indicate delayed second mRNA vaccine dose may suffice in previously infected individuals with RBD-directed immunological memory (39).The stable antibody levels observed post-booster dose, maintaining high responsiveness from initial vaccination, suggest the possibility of prolonged immune protection in this population, indicating that booster administration might be deferred to when antibody levels begin to wane. This observation aligns with debates in global health about the need and timing of booster doses.

One of the study’s primary limitations was the relatively small sample size, particularly for the S-IgG+ group, which limits the robustness of some inferences. Larger cohort studies in the SSA region would further solidify our findings. Additionally, as this study primarily focused on binding antibody responses, evaluating neutralizing antibodies across the diversity of strains in future studies would offer a more holistic view of vaccine-induced protection. Furthermore, it is critical to recognize the limitations of focusing primarily on antibody analysis. Notably, even in the absence of measurable antibody responses, T cell-mediated cellular immunity can continue. This phenomenon, in which lymphoma patients undergoing B-cell depleting therapy demonstrate CD8 T-cell responses with frequencies and magnitudes equivalent to healthy controls, emphasizes the crucial potential for powerful cellular immune responses despite a decreased serological profile (40, 41). Lastly, further research is necessary to optimize booster dose scheduling, especially as antibody levels remained elevated at the time of boosting in this setting. Future research should incorporate larger, more heterogeneous populations, and expanded immunological parameters, to furnish a more comprehensive picture.

Our study sheds light on the mRNA-1723 vaccine’s immunogenicity in a previously understudied SSA population, offering insights for a robust and prolonged immune response post-priming but a non-significant boost after the second dose. These findings highlight the importance of contextualizing vaccine strategies to specific demographics. The sustained S-IgG and S-IgA responses suggest the vaccine’s promise in conferring enduring protection, crucial for regions grappling with logistical and economic challenges of vaccination. The absence of marked changes in antibody levels following the booster dose could imply that a single dose might provide adequate protection over an extended period within this demographic. Alternatively, it might reflect a plateau in the immune response, where the initial vaccine dose already fully primes the immune system. This observation calls for a reassessment of the schedule for subsequent doses in this group. Further extensive studies incorporating larger cohorts and diverse immune markers are crucial to substantiate these findings and guide vaccination strategies tailored for the unique SSA demographic.

## Data availability statement

The raw data supporting the conclusions of this article will be made available by the authors, without undue reservation.

## Ethics statement

The studies involving humans were approved by the Research and Ethics Committee (GC/127/833) of the Uganda Virus Research Institute and the Uganda National Council for Science and Technology (HS637ES). The studies were conducted in accordance with the local legislation and institutional requirements. The participants provided their written informed consent to participate in this study.

## Author contributions

JS: Conceptualization, Data curation, Formal analysis, Funding acquisition, Investigation, Methodology, Project administration, Supervision, Visualization, Writing – original draft, Writing –review & editing. VA: Conceptualization, Data curation, Formal Analysis, Visualization, Writing – original draft, Writing – review & editing. JK: Data curation, Methodology, Validation, Writing –review & editing. CB: Data curation, Investigation, Methodology, Writing – review & editing. GKO: Investigation, Methodology, Writing – review & editing, Validation. GO: Data curation, Investigation, Methodology, Writing – review & editing. HN: Data curation, Methodology, Writing – review & editing, Investigation, Validation. SM: Data curation, Methodology, Writing – review & editing, Conceptualization. AN: Investigation, Methodology, Writing– review & editing, Data curation. IS: Investigation, Methodology, Writing – review & editing. PE: Investigation, Methodology, Writing– review & editing. LK: Investigation, Methodology, Writing – review & editing, Validation. TCIT: Investigation, Writing – review & editing, Methodology. MM: Investigation, Supervision, Writing – review & editing, Conceptualization. PK: Investigation, Supervision, Writing –review & editing, Validation.

## COVID-19 Immunoprofiling team

Jackson Sembera, Betty Oliver Auma, Solomon Opio, Ben Gombe.

## Supplementary material

Supplementary Figure 1 Boxplots illustrating the 12-month longitudinal trends of Nucleoprotein (N)-directed antibody optical densities (OD) and concentrations. **Supplementary Figure 1** depicts the nucleoprotein-directed antibody responses over 12 months after the first Moderna vaccine dose. Each box plot highlights the interquartile range, with the mean indicated by a solid black circle and the median by a horizontal line. The unpaired Wilcoxon test assessed differences in antibody responses across time points, with adjustments for multiple testing using the Hochberg test. The graph’s x-axis tracks the time elapsed since vaccination, using “PP” for periods after the primary vaccine dose (post-prime) and “PB” for periods following the booster dose (post-boost). The y-axis quantifies the optical densities and concentrations observed. We conducted a comparative analysis of significance between successive time points to assess the dynamic evolution of antibody responses throughout the study. This approach emphasizes the significance of changes observed between adjacent time points. As such, significant values are strategically positioned between time points, emphasizing their chronological relevance.

Supplementary Figure 2 Pairwise Differential Median Fold Changes in Nucleoprotein-Directed Antibodies and Number of Breakthrough Infections Across Sequential Time Points. illustrates the median fold-changes in Nucleoprotein-directed antibody levels across successive time points, with increases depicted in red and decreases in green (**Supplementary Figure 2A**). The fold change represents the ratio of median antibody levels between consecutive intervals, providing a clear visualization of the antibody trajectory. In assessing fold changes, In evaluating fold changes, a reference timepoint (on the y-axis) is evaluated against a subsequent timepoint (intersection on x-axis). A value of one within a box signifies no change from the baseline reference; values below one indicates a decrease, while values above one signifies an increase. **Supplementary Figure 2B** delineates the prevalence of presumed infection and breakthrough cases in the study cohort, measured by the change in N-IgG antibody levels, before and after completion of the COVID-19 vaccination regimen. Grey circles indicate the percentage of subjects presumed infected at each time point before completing the vaccination regimen, while black circles represent the percentage of breakthrough cases post-full vaccination. The y-axis quantifies these percentages. Breakthrough cases, defined as subjects with an 11-fold increase in N-IgG levels indicative of infection occurring 14 days or more after the complete vaccination, amounted to three individuals, all of whom were identified six months post-vaccination. The “COVID-19 Immunoprofiling team” is listed as follows; Jackson Sembera, Betty Oliver Auma, Solomon Opio, Ben Gombe.
