# Supplementary material for: Sustained S-IgG and S-IgA antibodies to Moderna’s mRNA-1273 vaccine in a Sub-Saharan African cohort suggests need for booster timing reconsiderations
Source: Front Immunol. 2024 Jan 31;15:1348905. doi: 10.3389/fimmu.2024.1348905 (PMC10864610; doi:10.3389/fimmu.2024.1348905)
Supplement: Supplementary Figure 1 — Boxplots illustrating the 12-month longitudinal trends of Nucleoprotein (N)-directed antibody optical densities (OD) and concentrations. Supplementary Figure 1 depicts the nucleoprotein-directed antibody responses over 12 months after the first Moderna vaccine dose. Each box plot highlights the interquartile range, with the mean indicated by a solid black circle and the median by a horizontal line. The unpaired Wilcoxon test assessed differences in antibody responses across time points, with adjustments for multiple testing using the Hochberg test. The graph’s x-axis tracks the time elapsed since vaccination, using “PP” for periods after the primary vaccine dose (post-prime) and “PB” for periods following the booster dose (post-boost). The y-axis quantifies the optical densities and concentrations observed. We conducted a comparative analysis of significance between successive time points to assess the dynamic evolution of antibody responses throughout the study. This approach emphasizes the significance of changes observed between adjacent time points. As such, significant values are strategically positioned between time points, emphasizing their chronological relevance. [file Presentation_1.pdf]

Supplementary Figures

Supplementary Figure 1: Boxplots illustrating the 12-month longitudinal trends of Nucleoprotein (N)-directed antibody optical densities (OD) and concentrations.

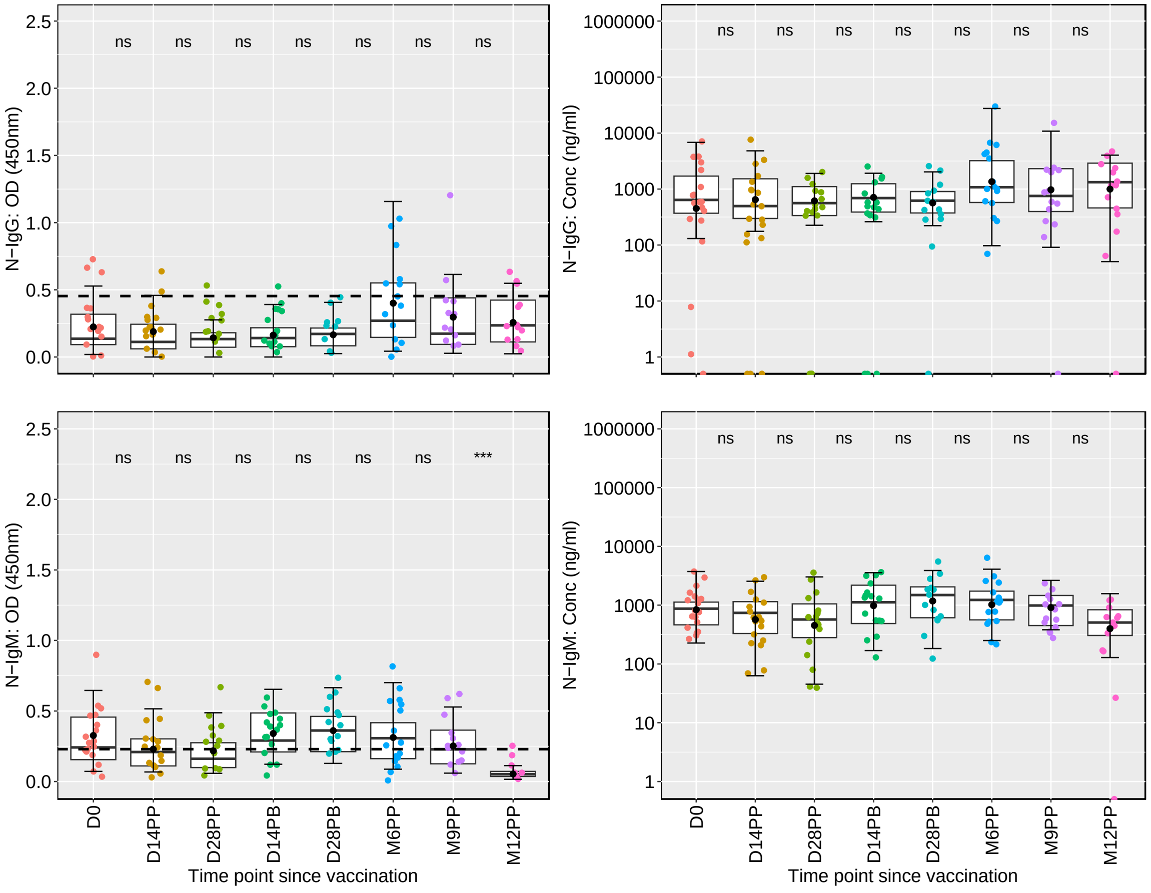

Supplementary Figure 1 depicts the nucleoprotein-directed antibody responses over 12 months after the first Moderna vaccine dose. Each box plot highlights the interquartile range, with the mean indicated by a solid black circle and the median by a horizontal line. The unpaired Wilcoxon test assessed differences in antibody responses across time points, with adjustments for multiple testing using the Hochberg test. The graph's x-axis tracks the time elapsed since vaccination, using "PP" for periods after the primary vaccine dose (post-prime) and "PB" for periods following the booster dose (post-boost). The y-axis quantifies the optical densities and concentrations observed. We conducted a

comparative analysis of significance between successive time points to assess the dynamic evolution of antibody responses throughout the study. This approach emphasizes the significance of changes observed between adjacent time points. As such, significant values are strategically positioned between time points, emphasizing their chronological relevance. Significance levels are indicated as not significant (ns) for $p > 0.05$ and *** for $p < 0.001$.

Supplementary Figure 2: Pairwise Differential Median Fold Changes in Nucleoprotein-Directed Antibodies and Number of Breakthrough Infections Across Sequential Time Points

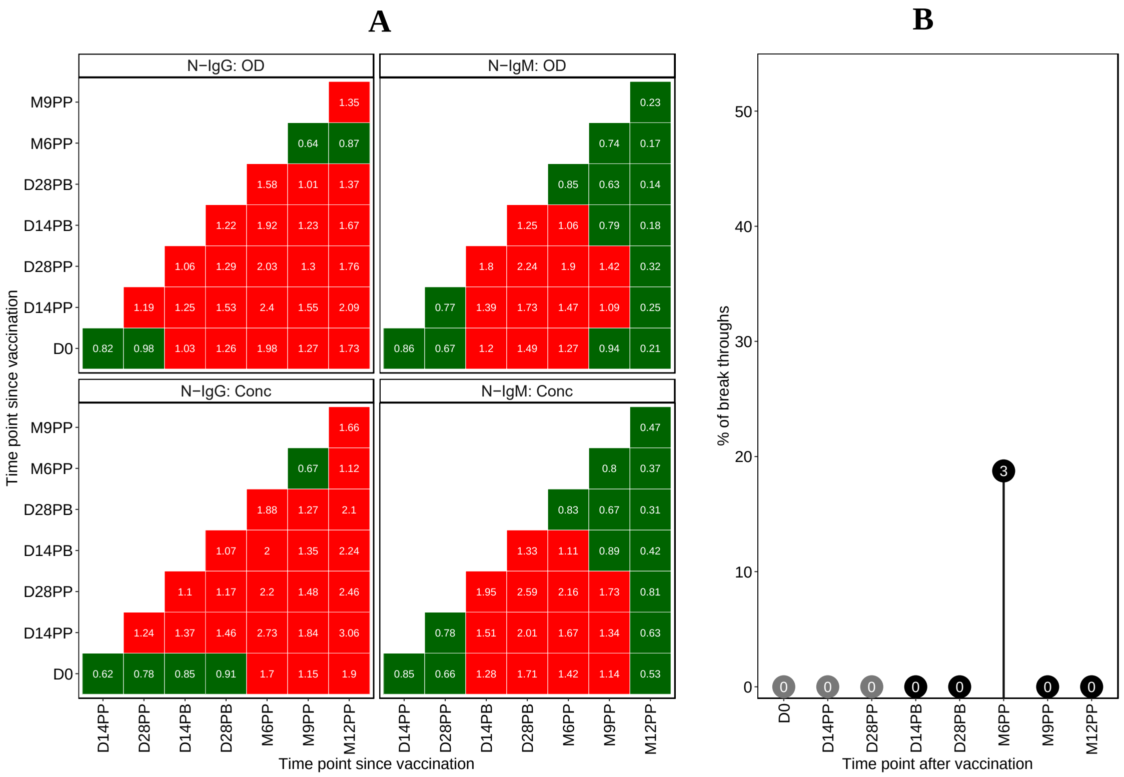

Supplementary Figure 2 illustrates the median fold-changes in Nucleoprotein-directed antibody levels across successive time points, with increases depicted in red and decreases in green (Supplementary Figure 2A). The fold change represents the ratio of median antibody levels between consecutive intervals, providing a clear visualisation of the antibody trajectory. In evaluating fold changes, a reference timepoint (on the y-axis) is evaluated against a subsequent timepoint (intersection on x-axis). A value of one within a box signifies no change from the baseline reference; values below one indicates a decrease, while values above one signifies an increase. Supplementary Figure 2B delineates the prevalence of presumed infection and breakthrough cases in the study cohort, measured by the change in N-IgG antibody levels, before and after completion of

the COVID-19 vaccination regimen. Grey circles indicate the percentage of subjects presumed infected at each time point before completing the vaccination regimen, while black circles represent the percentage of breakthrough cases post-full vaccination. The y-axis quantifies these percentages. Breakthrough cases, defined as subjects with an 11-fold increase in N-IgG levels indicative of infection occurring 14 days or more after the complete vaccination, amounted to three individuals, all of whom were identified six months post-vaccination.
